# The Association between Different Levels of Alcohol Use and Gait under Single and Dual Task in Community-Dwelling Older Persons Aged 65 to 70 Years

**DOI:** 10.1155/2016/2018507

**Published:** 2016-07-19

**Authors:** Laurence Seematter-Bagnoud, Christophe Büla, Brigitte Santos-Eggimann

**Affiliations:** ^1^Institute of Social and Preventive Medicine, Route de la Corniche 10, 1010 Lausanne, Switzerland; ^2^Service of Geriatric Medicine and Geriatric Rehabilitation, Department of Medicine, Lausanne University Hospital, Rue du Bugnon 46, 1011 Lausanne, Switzerland

## Abstract

*Objectives*. This study aimed to describe the cross-sectional and longitudinal association between alcohol intake and gait parameters in older persons.* Methods*. Community-dwelling persons aged 65–70 years (*N* = 807). Information on health, functional status, and alcohol use was self-reported at baseline and at 3-year follow-up, whereas gait speed and stride-to-stride variability were measured while walking only (single task) and under dual tasking (counting backwards).* Results*. Compared to light-to-moderate drinking, heavy drinking was associated with slower gait speed in single task (adj. coeff.: −.040, 95% CI: −.0.78 to −.002, *p* = .035). No significant association was observed between heavy drinking and gait speed variability. Nondrinkers walked significantly slower than light-to-moderate drinkers in dual task and had significantly higher gait speed variability in both single and dual task, but these associations disappeared after adjustment for comorbidity. At follow-up, 35.2% and 34.1% of the participants walked significantly slower in single and dual task, respectively. This proportion varied a little across drinking categories.* Conclusion*. At baseline, heavy alcohol consumption was significantly associated with slower gait speed in single task. Selective survival of the fittest heavy drinkers probably explains why this association faded in longitudinal analyses. The trend of poorer gait performance in nondrinkers disappeared after adjustment for comorbidity, suggesting confounding by a worse health status.

## 1. Introduction

The consequences of chronic alcohol abuse on gait and balance are well known, but the effects of moderate consumption on gait parameters still received little attention. Some previous studies observed that even moderate alcohol intake might affect cerebellar cells over the long term, causing ataxia and increased body sway, which may affect gait [1–3]. However, the relationship between alcohol-related cerebellar damage and gait speed and variability remains controversial. For instance, a study performed among older adults failed to demonstrate any significant association between alcohol-related reduced cerebellar volume and features of ataxic gait [2].

Even though moderate alcohol use might not cause gross alteration of usual gait, the effect of alcohol might become more obvious when stressors are added, such as walking under dual task condition. Indeed, gait is a highly complex, semiautomatic motor function, which requires some amount of attentional resources [4, 5]. It has been shown that gait slows under dual task condition, with increased gait variability [5]. This effect is more pronounced when cognition is impaired, showing the inability to allocate attention properly when performing a simultaneous cognitive task, such as counting backward while walking [5]. As alcohol also causes cortical brain damage, dual tasking might reveal subtle negative consequences of alcohol use in subjects who drink beyond recommended threshold, but who are not alcoholics.

On the other hand, there is some evidence that light-to-moderate alcohol use has a protective effect against vascular diseases [6]. One could hypothesize that it could therefore balance the potential detrimental influence of alcohol on gait, by preventing damage to cerebral circulation.

Despite the high prevalence of both gait impairment and alcohol use in older persons, we found no study that specifically investigated this relationship in this specific population. This analysis aimed to examine the cross-sectional and prospective association between different levels of alcohol use and gait parameters. The hypothesis was that drinking above recommended threshold would be associated with poorer gait performance measured over a 20-meter walk, as compared to moderate drinking that might have a protective effect. Specifically, we expected that higher alcohol consumption would be associated with slower gait speed and higher gait speed variability. The negative influence of higher alcohol intake on gait was expected to be more evident under cognitive dual task condition. In addition, we hypothesized that controlling for comorbidity will attenuate the observed associations.

## 2. Methods

The design of the Lausanne cohort 65+ study has been previously described [7]. Briefly, this population-based cohort launched in 2004 enrolled 1564 randomly selected community-dwelling persons aged 65 to 70 years, living in the city of Lausanne, Switzerland.

### 2.1. Data Collection

In 2004, participants completed a questionnaire that included data about demographics, education, lifestyle habits, health, and functional status. Alcohol use was measured using the AUDIT-C [8]. In 2005, participants underwent a face-to-face interview with baseline physical examination as well as physical and cognitive performance tests, including gait analysis in a subsample of 807 participants (61.6% of the 1310 participants). The main reasons for missing gait parameters were unavailability of the recording device (64.8%), inability to walk due to health and/or safety problems (20.8%), and refusal (3.6%). Participants who underwent gait recording were more frequently men, were married, had higher education and better functional status, and reported fewer falls than those who did not (*data not presented*).

In 2008, follow-up assessment of gait performance was available in 684 (84.7%) of the 807 participants. Deaths were ascertained using the local population register.

### 2.2. Operationalization of the Alcohol Variable

The average number of standard drinks (wine, beer, and spirits) consumed per week was estimated using the first and second questions of the AUDIT-C. As described previously [9], alcohol intake was categorized into “none,” “light-to-moderate,” “at-risk,” and “heavy.” “Light-to-moderate” drinking was defined according to the usual recommendation of maximum of 1 drink per day in women and 2 drinks per day in men [10]. The threshold separating “at-risk” from “heavy” drinking was defined based on cut-offs used in similar studies, with at-risk drinking corresponding to 8–11 drinks per week in women and 15–19 drinks per week in men and heavy drinking encompassing any intake above these limits [11].

### 2.3. Gait Assessment

Gait parameters were measured over 20 meters in a well-lit walkway, under the same conditions at baseline and 3-year follow-up. Participants were asked to walk at self-selected, usual, speed as when they go out to do shopping, for example. During dual task, participants were asked to count backwards aloud from fifty while walking. No instruction was given regarding prioritization of any task. Each test was performed once, except when the test failed due to a misunderstanding of the instructions or a technical problem. Gait speed (m/s) was estimated from the angular velocity recorded by the Physilog® system, a device that includes 4 body-fixed sensors on lower limbs and a data logger carried on the waist (BioAGM, Tour-de-Peilz, Switzerland) [12]. Within person gait speed variability was assessed using the coefficient of variation (CV in %) defined as the standard deviation divided by the mean value of gait speed for each stride.

### 2.4. Statistical Analyses

Characteristics of participants (including gait parameters) were compared across the categories of alcohol intake, using Chi-square test for categorical variables. Kruskal-Wallis test was used for continuous variables because of nonnormal distribution and the presence of outliers.

Robust linear regression analyses were performed to examine the cross-sectional association between alcohol intake and gait parameters to take heteroscedasticity into account, both in bivariate and in multivariate models. As men and women displayed different patterns of alcohol consumption and gait parameters, a gender-alcohol interaction was also systematically tested in multivariate models. Other covariates were included in the analyses based on their association with both alcohol consumption and gait speed: age, education (with low education defined as less than 12 years of education), and comorbidity (defined as self-reporting 2 or more medical diagnoses out of the following list: hypertension, coronary heart disease, other heart diseases, stroke, diabetes mellitus, chronic respiratory disease, arthritis, osteoporosis, gastrointestinal ulcer, depression, Parkinson disease, and cancer).

The multivariate model also included abnormal cognition (MMSE < 24), as cognition has been shown to significantly influence gait performance.

Bivariate analyses examined the prospective association between baseline alcohol use and three-year changes in gait speed and its CV under single and dual task conditions, respectively. Regarding gait speed, the outcome was defined as a decline of 0.1 m/s, which is considered as a clinically meaningful change [13]. Analyses were conducted using Stata, version 13.0.

The study was approved by the Cantonal Human Research Ethical Committee (protocol 64/14, decision: March 11, 2014), and written consent was obtained from all participants during the in-person visit.

## 3. Results

Overall, 91.3% of the participants (mean age: 67 years, 55% women, 46% reporting more than one chronic disease, and 7% reporting any impairment in basic activities of daily living) reported consuming some alcohol over the previous 12 months. Among those, two-thirds reported light-to-moderate drinking, about a fourth reported at-risk drinking, and a tenth reported heavy drinking (Table 1). Comparisons of baseline characteristics across drinking categories showed that nondrinkers and heavy drinkers were twice as likely as moderate and at-risk drinkers to report functional impairment (14.1%, and 10.8%, resp., versus 6.1% and 7.7% among moderate and at-risk drinkers, *p* = .073). Nondrinkers more frequently reported comorbidity (59.2% versus 40% to 45% in other categories, *p* = .067) and were more often cognitively impaired (5.7% versus <2% in other categories, *p* = .001, defined as a Minimental State Examination score <24/30 (11)).

### 3.1. Baseline Gait Analysis

Overall, gait speed was 1.13 ± 0.16 m/s under single task and decreased to 0.99 ± 0.19 m/s in dual task (counting backwards). Average gait speed variability in the entire sample was 3.5% under single task condition and increased up to 6.2% during dual task. Comparison across categories indicated slower gait speed and higher gait speed variability among nondrinkers, as well as among heavy drinkers, but these differences were not significant in bivariate analysis (Table 1).

During dual task, the proportion of participants counting backwards without any error was 63.3%, 76.9%, 83.4%, and 76.1% among nondrinkers, low-to-moderate drinkers, at-risk and heavy drinkers, respectively (*p* = .052). The mean number of errors did not differ significantly across categories of alcohol intake.

Table 2 shows the results of the crude and adjusted models examining the cross-sectional relationship between gait parameters and alcohol intake, using the light-to-moderate drinkers as the reference group. In bivariate analyses, the most consistent pattern was observed among nondrinkers who showed slower gait speed and increased gait variability under both single and dual task conditions. However, these differences did not remain once controlling for comorbidity in multivariate analyses. Although heavy drinkers also presented with similarly altered gait pattern, only gait speed under single task condition was significantly reduced (adjusted coeff.: −.040, 95% CI: −.0.78 to −.002, *p* = .035).

### 3.2. Longitudinal Analysis

About 16% of subjects had no follow-up gait recording, half of them because health-related reasons precluded the performance tests. As compared to participants with follow-up measurements, they had slower gait speed at baseline (1.07 ± .17 m/s versus 1.14 ± .15 m/s, *p* < .001), as well as higher speed variability (4.1% versus 3.4%, *p* = .003). Over the three years of follow-up, 39 subjects died, corresponding to 8.1% of heavy drinkers versus only 2.8% of nondrinkers (*p* = .496). When comparing baseline and 3-year gait performance, a clinically significant decline in speed (≥0.10 m/sec) was observed in 35.2% of the subjects under single task condition. This proportion did not differ across levels of alcohol use (range: 32.8% (heavy drinkers) to 37.7% (nondrinkers), *p* = .690). Speed decline occurred in a similar proportion (34.1%) of participants under dual task condition. Again, no significant association was observed across drinking categories.

**Figure 1:**
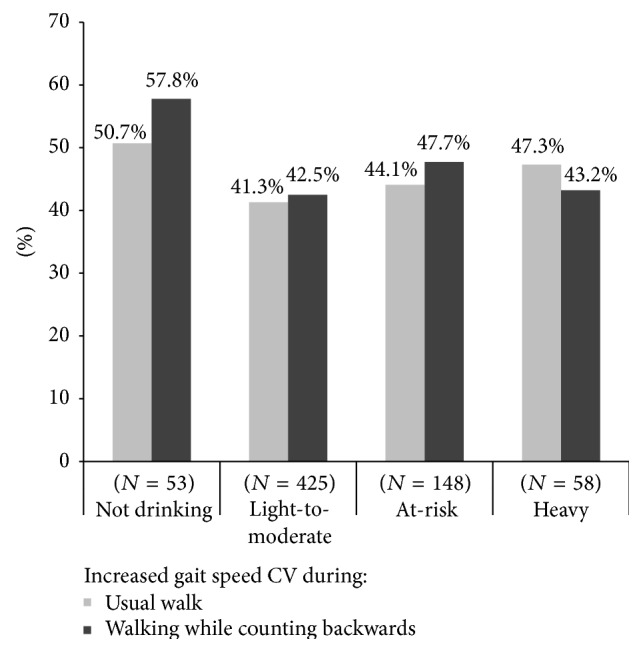
Proportion of participants with increased gait speed variability at 3-year follow-up as compared to baseline, according to alcohol intake and walking condition.

Gait variability also deteriorated (increase) at 3-year follow-up in 43.3% and 45.0% of participants under single and dual task condition, respectively.  Figure 1 displays the proportions of participants in each drinking category with increased gait speed variability at follow-up under single and dual task conditions, respectively. These proportions were highest in nondrinkers and lowest among light-to-moderate drinkers and increased progressively as alcohol consumption increased. This U-shaped relationship was most apparent in single task condition but was not statistically significant.

## 4. Discussion

Results from cross-sectional and longitudinal analyses in this large sample of community-dwelling elderly indicate that alcohol abstention as well as excessive use were associated with poorer gait performance (slower gait speed and increased gait variability), suggesting a U-shaped relationship. However, these associations were inconstant and varied according to adjustment for health variables. Several factors might contribute to the failure of finding an independent effect of alcohol on gait measurement.

First, both nondrinkers and heavy drinkers differ from moderate drinkers in terms of health status. A previous analysis on a subgroup of abstinent participants (*n* = 70) indicated that half were ex-drinkers, most of whom stopped drinking because of health problems, while the other half were never drinkers who also had a poorer health status than moderate drinkers [9]. As this information was available for this small subsample only, it precluded its use for further analyses.

Second, the small number of heavy drinkers (*N* = 58) resulted in limited statistical power, especially in longitudinal analyses. As heavy drinkers are less likely to participate in research study on health, the proportion observed in the current study probably underestimates their true prevalence. However, this proportion is very similar to those observed in other studies undertaken in Switzerland [14]. In addition, results from the longitudinal analyses were probably biased by selective attrition of the less healthy participants and a healthy survivor effect: results showing worse gait performance at baseline (slower gait speed and increased gait variability) among participants lost to follow-up, as well as the increased death rate among heavy drinkers, support this hypothesis of a healthy survivor effect. This likely resulted in underestimation of the deleterious effect of heavy alcohol intake on gait.

Finally, although cognitive impairment has been shown to be associated with slower and more irregular gait speed [5], the very low proportion of participants with cognitive impairment as assessed through an abnormal Minimental State Examination in our study precluded examining this association in more depth. Perhaps using more detailed neuropsychological tests focusing on executive functioning might have been useful in this context.

Another interesting finding from this study is the relatively small proportion (about a third for gait speed and a half for gait variability) of participants who had worse gait performance at 3-year follow-up. This observation extends previous observation showing that gait speed remains relatively constant throughout adult life until age of 65 where it starts to decline by 1-2% per year (.008 m/s to .03 m/s) up to 80 years [15–17]. In this context, the use of the Physilog device was useful to detect these subtle changes in gait speed and variability.

This low proportion of participants with decline in gait performance could also result from the previously mentioned healthy survivor effect. In addition, while participants were relatively young and fit, their average gait speed at baseline was at the lower end of age-specific normative values previously reported in community-dwelling older persons [18, 19].

Finally, an original contribution of this study is to show that dual tasking did not improve the detection of gait differences across drinking categories. This was contrary to the hypothesis that excessive drinking would be associated to slower and more irregular gait, partly through poorer executive performance caused by excessive drinking [5, 20]. In addition to factors previously discussed, the selection of the dual task could explain this negative finding. Perhaps by selecting a more complex and challenging cognitive task than counting backward, it might have provided different results.

In conclusion, a negative association between heavy alcohol consumption and gait speed was significant in cross-sectional analysis and under single task only. Healthy survival effect probably explains why this association was not retrieved in longitudinal analyses. The observed trend of poorer gait performance in nondrinkers disappeared after adjustment for comorbidity, suggesting confounding by a worse health status in this group. Even though this work did not show significant negative effects of excessive alcohol consumption on gait performance, these results do not mean that older persons should be encouraged to drink. In particular, occasional heavier drinking confers an increased risk for falls and accidents. Globally, the risk associated with drinking above recommended thresholds may well outweigh any potential benefit for many older persons.

## Figures and Tables

**Table 1:** Characteristics of participants and comparison according to alcohol intake.

	Total	Drinking groups				*p* value^*∗*^
		Not drinking	Light-to-moderate	At-risk	Heavy	
	*N* = 807	*N* = 71	*N* = 492	*N* = 170	*N* = 74	
	100%	8.8%	61.0%	21.1%	9.2%	
Age (mean ± SD)	67.0 ± 1.4	67.2 ± 1.4	67.0 ± 1.4	66.8 ± 1.3	67.0 ± 1.4	.221
Men (%)	46.3	25.4	44.1	53.5	64.9	<.001
Low education (%)^†^	64.2	70.4	62.3	65.7	67.6	.480
Comorbidity (2^+^ chronic diseases, %)^‡^	46.2	59.2	45.5	45.0	40.5	.001
Cognitive impairment (%)^§^	1.9	5.7	1.6	1.8	0.0	.067
Instrumental ADLs impairment (%)^||^	10.5	16.9	8.5	12.9	12.2	.094
Basic ADLs impairment (%)^||^	7.6	14.1	6.1	7.7	10.8	.073
Fear of falling (%)	37.5	45.4	37.0	35.0	39.1	.508
Any fall over the past 12 months (%)	16.5	23.9	17.4	9.6	18.8	.034
Single task: walking at usual speed						
(i) Gait speed (m/s, mean ± SD)	1.13 ± .16	1.10 ± 0.17	1.14 ± 0.15	1.14 ± 0.15	1.11 ± 0.18	.194
(ii) Gait speed CV^*∗*^ (%)	3.52	3.58	3.44	3.30	3.64	.410
Dual task: walking while counting backwards						
(i) Gait speed (m/s, mean ± SD)	0.99 ± 0.19	0.94 ± 0.20	1.00 ± 0.19	1.00 ± 0.18	0.97 ± 0.20	.061
(ii) Gait speed CV^*∗*^ (%)	6.22	6.68	6.09	5.93	6.48	.256

^*∗*^ *p* value from Chi-square test (categorical variables) or ANOVA (continuous variables).

^†^Defined as less than 12 years of education (compulsory school or apprenticeship).

^‡^Defined as self-reporting 2 or more conditions out of the following list: hypertension, coronary heart disease, other heart diseases, stroke, diabetes mellitus, chronic respiratory disease, arthritis, osteoporosis, gastrointestinal ulcer, depression, Parkinson disease, and cancer.

^§^Defined as a score <24/30 at Folstein's Minimental State Examination.

^||^Instrumental activities of daily living include shopping and performing usual household activities. Basic activities of daily living were bathing, dressing, using the toilet, transferring from and to bed or chair, and feeding.

**Table 2:** Cross-sectional association of gait parameters and alcohol intake^*∗*^.

	Bivariate analysis^*∗∗*^			Multivariate analysis^*∗∗∗*^		
	Coefficient	95% CI	*p* value	Coefficient	95% CI	*p* value
Single task: walking at usual speed						
*Gait speed (m/s, positive coefficient indicating higher speed)*						
No drinking	−.035	−.073–.004	.077	−.026	−.065–.013	.193
Light-to-moderate drinking	*Reference*			*Reference*		
At-risk drinking	.002	−.025–.029	.899	−.002	−.029–.026	.907
Heavy drinking	−.032	−.069–.006	.103	−.040	−.078–−.002	.035
*Gait speed CV (%, positive coefficient indicating greater gait speed variability)*						
No drinking	.337	.048–.626	.022	.288	−.017–.581	.064
Light-to-moderate drinking	*Reference*			*Reference*		
At-risk drinking	−.030	−.023–.017	.770	−.006	−.022–.020	.955
Heavy drinking	−.083	−.037–.200	.564	.008	−.029–.300	.954

Dual task: walking while counting backwards						
*Gait speed (m/s)*						
No drinking	−.053	−.101–−.004	.031	−.032	−.081–.015	.113
Light-to-moderate drinking	*Reference*			*Reference*		
At-risk drinking	.010	−.024–.043	.563	.01	−.021–.040	.462
Heavy drinking	−.025	−.072–.021	.285	−.039	−.082–.017	.203
*Gait speed CV (%)*						
No drinking	.621	.004–1.24	.048	.475	−.017–1.12	.152
Light-to-moderate drinking	*Reference*			*Reference*		
At-risk drinking	.029	−.400–.458	.893	.013	−.467–.441	.952
Heavy drinking	.420	−.177–1.02	.168	.418	−.210–1.04	.192

^*∗*^As separate models for men and women had close results and the test for interaction was not significant, a unique model is displayed in the table.

^*∗∗*^Results from bivariate robust regression.

^*∗∗∗*^Results from robust regression model adjusting for age, gender, education, previous falls, comorbidity, and cognition.
